# Additive Therapy of Plasmodium berghei-Induced Experimental Cerebral Malaria via Dihydroartemisinin Combined with Rapamycin and Atorvastatin

**DOI:** 10.1128/spectrum.02317-22

**Published:** 2023-03-22

**Authors:** Xiaonan Song, Weijia Cheng, Huiyin Zhu, Yuting Li, Jian Li

**Affiliations:** a School of Basic Medical Sciences, Hubei University of Medicine, Shiyan, China; National Institutes of Health

**Keywords:** Additive therapy, atorvastatin, cerebral malaria, combination therapy, dihydroartemisinin, rapamycin

## Abstract

Cerebral malaria (CM), caused by Plasmodium falciparum, is the primary cause of death from severe malaria. Even after immediate parenteral therapy with antimalarial drugs, the mortality rate remains 15 to 25%. Currently, no effective therapeutic agents are available for the radical treatment of CM. Thus, further in-depth explorations of adjuvant therapies in combination with antimalarial drugs are urgently needed. The experimental cerebral malaria (ECM) model was established by infecting C57BL/6 mice with Plasmodium berghei ANKA. Subsequently, infected mice were continuously treated with dihydroartemisinin (DHA) in combination with rapamycin (RAP) and atorvastatin (AVA) for 5 days at different time points, including day 0, day 3, and day 6 postinfection (p.i.). Treatment efficacy was evaluated by comparing behavioral scores, body weight, parasitemia, survival rate, blood-brain barrier (BBB) integrity, and histopathology. The optimal combination therapy of DHA, RAP, and AVA on day 3 p.i. was selected for ECM. This strategy significantly improved survival rate, reduced parasitemia, improved the rapid murine coma and behavioral scale scores and permeability of the BBB, attenuated cerebrovascular and hepatic central venous obstruction and hemozoin deposition in the liver, and decreased the red pulp area of the spleen, which effectively ameliorated neurological damage in ECM. It also improved histopathology and neurological damage caused by ECM. In this study, the optimal therapeutic strategy for ECM was selected, which is expected to be a potential therapy for human CM.

**IMPORTANCE** Although artemisinin-based combination therapies (ACTs) have greatly improved the clinical outcome of cerebral malaria (CM) as a fatal disease that can permanently disable a significant proportion of children even if they survive, new treatment options are needed as Plasmodium falciparum develops resistance to antimalarial drugs. Recent reports suggest that basal treatment with artemisinin derivatives often fails to protect against cell death, neurological damage, and cognitive deficits. In this study, the combination of dihydroartemisinin with rapamycin and atorvastatin improved the current antimalarial outcomes by overcoming the limitations of current antimalarials for CM morbidity and neurological sequelae. This combination offers a new adjunctive treatment for the clinical treatment of human CM in susceptible populations, including children under 5 years old and pregnant women.

## INTRODUCTION

Malaria remains one of the most severe infectious diseases worldwide. In 2021, the estimated number of malaria cases rose to 247 million cases, and malaria deaths increased to an estimated 619,000, a slight decline compared with 2020 (1). As a fatal complication of malaria, cerebral malaria (CM) caused by Plasmodium falciparum (*P. falciparum*) is the most frequent and serious parasitosis that influences the human central nervous system (2). It is estimated that approximately 2% of the population infected with P. falciparum develops a more severe form of malaria, which may eventually lead to cerebral complications, particularly CM (3). Human CM generally occurs in children aged under 5 years, pregnant women, and individuals with low immunity. Clinical symptoms of CM include coma, seizures, and hypoglycemia (4). In addition, a proportion (1/3) of survivors suffer lifelong neurological impairment (5) related to antimalarial drugs that are effective in clearing parasites but do not necessarily prevent damage to the brain parenchyma.

Drug therapies are currently the primary therapy for malaria patients. However, the use of monotherapies for several decades has led to the development of resistance to several excellent antimalarial drugs (6). This not only surmounts the problem of drug resistance caused by a single drug but also effectively alleviates the global epidemic of malaria. Artemisinin (ART) and its derivatives are currently the first-line antimalarial drugs for the treatment of CM (7). The occurrence of antimalarial drug resistance is constantly increasing (8). Moreover, antimalarial therapy alone cannot comprehensively improve neurological and brain pathological damage (9). Therefore, because combination therapy has been shown to be efficacious against other diseases, there is an urgent need to explore combination treatments with additive effects with ART to reduce or prevent the occurrence of CM.

Mammalian target of rapamycin (mTOR), a serine/threonine protease, plays a regulatory role in immune homeostasis by integrating different response signals from the internal microenvironment (10). In the context of experimental cerebral malaria (ECM), evidence suggests that rapamycin (RAP) treatment confers significant protection against ECM symptoms and prevents ECM-related pathological damage (11). Statins, inhibitors of 3-hydroxy-3-methylglutaryl-coenzyme A reductase (HMG-CoA reductase) (12, 13), have lipid-lowering activity and are commonly used clinically to treat cardiovascular diseases (14, 15). In addition, atorvastatin (AVA) has also shown significant pleiotropic effects independent of its hypolipidemic activity, such as neuroprotective (16, 17) and anti-inflammatory (18) activities and endothelial recovery (19). Previous studies have demonstrated that AVA alone does not prevent death from ECM (20), but AVA in combination with antimalarials has an additive therapeutic effect on ECM (21, 22). Thus, AVA is considered an outstanding adjuvant in the treatment of CM (23).

This study adopted a two-pronged treatment strategy using dihydroartemisinin (DHA) combined with RAP and AVA for the treatment of ECM and the mode of administration at different time points. By comparing their phenotypic behaviors and histopathologies, we clarified whether the combination of the three drugs might additionally improve symptoms and have obvious advantages in neuroprotection in mice with ECM. This study thus was an exploration of a possible strategy for additive combination therapy of CM.

## RESULTS

### Effect on body weight and neurological signs in ECM.

**FIG 1:**
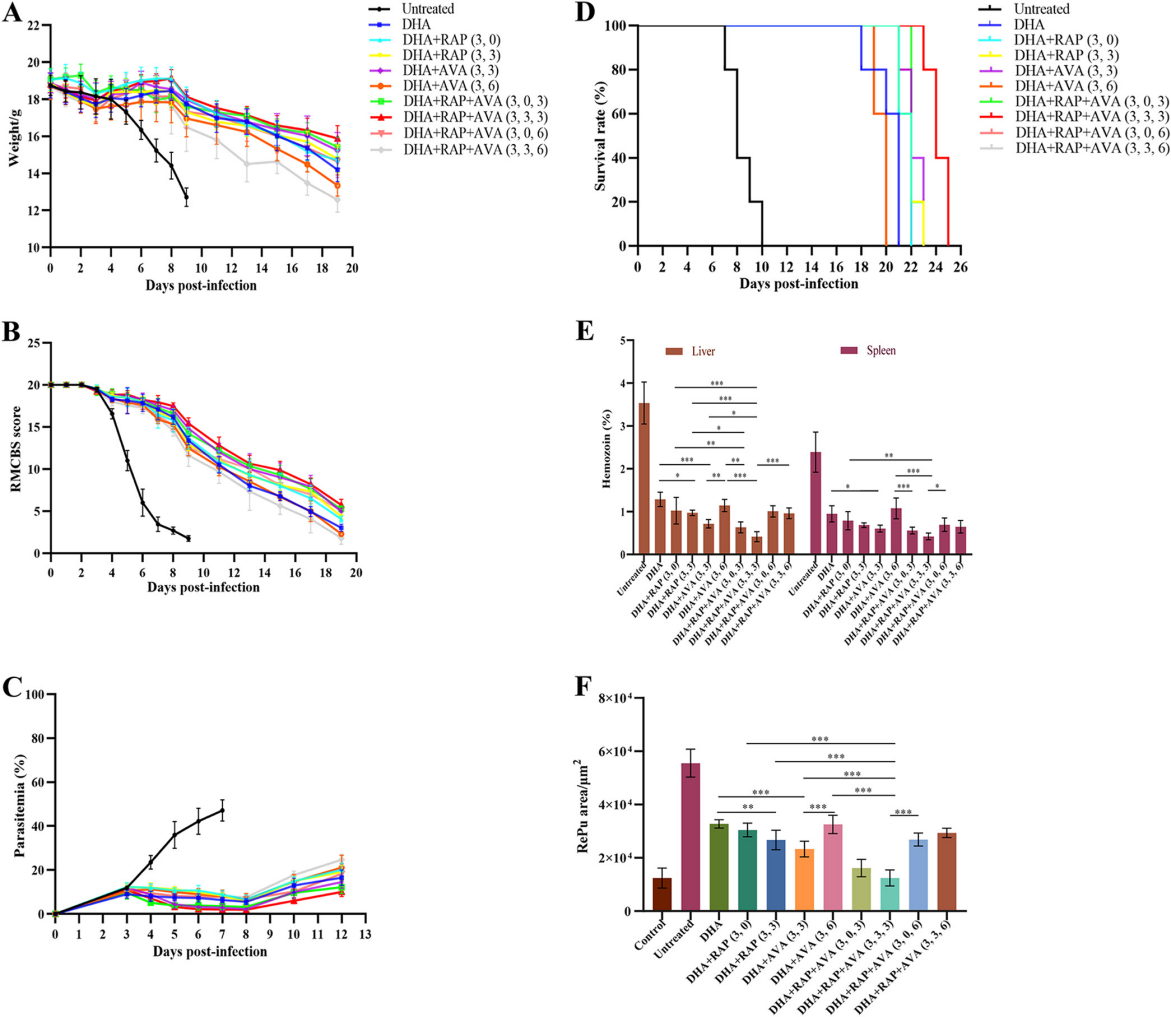
Monitoring of indicators associated with the ECM model receiving different treatments. (A) Body weight of ECM mice receiving different treatments; (B) RMCBS score curve of ECM mice receiving different treatments; (C) parasitemia in ECM mice receiving different treatments; (D) survival rate curve analysis of ECM mice; (E) analysis of the hemozoin proportion in the liver and spleen in the different treatment groups; (F) quantification of the red pulp (RePu) area for the treatment groups. The statistical analysis method was one-way ANOVA and the LSD posttest was used for each one-way ANOVA. Survival rate analysis was assessed by the log-rank (Mantel-Cox) test. The error bars indicate standard deviation. *, *P < *0.05; **, *P < *0.01; ***, *P < *0.001.

In combination therapy, except for the untreated group, the body weights of mice in all treated groups showed an overall first decreasing, then increasing, and decreasing trend from day 0 postinfection (p.i.) (Fig. 1A). On day 9 p.i., the weight of untreated mice was less than 13 g. In the double-drug combination treatment, the average body weight of the DHA+RAP (3, 0) group (see “Experimental grouping and drug treatment scheme” in Materials and Methods) was higher than that of the DHA+RAP(3,3) group for the combined treatment with the two drugs, but there was no significant difference between them. The DHA+RAP(3,3) group gained more weight on average than the DHA+AVA(3,6) group, and this difference was statistically significant (*P = *0.037). Among the triple-drug combination groups, the body weight was highest in the DHA+RAP+AVA(3,3,3) group and lowest in the DHA+RAP+AVA(3,6) group, and the difference between them was significant (*P = *0.001). However, the DHA+RAP+AVA(3,3,3) group was not significantly different from the other groups. There were no statistically significant differences in the DHA+RAP+AVA(3,3,3) group compared with the DHA+RAP (3, 0), DHA+RAP(3,3), and DHA+AVA(3,3) groups (Fig. 1A). In supplemental monotherapy, the body weight of the mice in each group increased and then decreased after infection (see Fig. S1A in the supplemental material). During the first 2 days p.i., the mice had not yet shown signs of disease, so their body weight increased slightly. On day 3 p.i., the mice tended to lose weight continuously because Plasmodium falciparum-infected red blood cells (iRBCs) began to increase in their bodies. On day 9 p.i., the body weight of the drug-treated mice was higher than that of the untreated mice, but the mean body weight in the four groups of drug-treated mice was not significantly different from that of the untreated group. Although the mean body weight was slightly higher in the RAP (0) group than in the RAP (3) group, there was no significant difference between the two groups. The average body weight of the AVA (3) group was higher than that of the AVA (6) group, but there was no significant difference between the two groups. On day 11 p.i., there was no significant difference in body weight between the untreated mice and the RAP- or AVA-treated mice (Fig. S1A).

In combination therapy, all groups of mice showed a decreasing trend in rapid murine coma and behavioral scale (RMCBS) scores (Fig. 1B). Untreated mice began to manifest symptoms of decreased exploration ability, weakened limb strength, and unsteady walking on day 4 p.i. On day 5 p.i., symptoms of CM started to appear, and the RMCBS scores declined rapidly. By day 10 p.i., the RMCBS score reached 1.75. In contrast, in the same period, treated mice showed distinctly higher RMCBS scores (Fig. 1B). From day 4 to day 8 p.i., the RMCBS scores of the treated mice decreased slightly but remained within the normal score range. The RMCBS scores of the DHA+RAP+AVA(3,3,3), DHA+RAP+AVA (3, 0, 3), and DHA+AVA(3,3) groups were stable. On day 9 p.i., the RMCBS score of the DHA+RAP (3, 0) group was higher than that of the DHA+RAP(3,3) group, but there was no significant difference between the two groups. The average score of DHA+AVA(3,3)-treated mice was significantly higher than that of DHA+AVA (3, 6)-treated mice (*P < *0.001). Among mice treated with DHA combined with RAP and AVA, RMCBS scores were highest in those treated with DHA+RAP+AVA(3,3,3). The DHA+RAP+AVA(3,3,3) combination was significantly different from DHA+RAP+AVA (3, 0, 3) (*P = *0.047), DHA+RAP+AVA (3, 0, 6) (*P < *0.001) and DHA+RAP+AVA(3,3,6) (*P < *0.001). For the triple-drug combination compared to the double-drug combination, the score of DHA+RAP+AVA(3,3,3) was better than those of DHA+RAP (3, 0) (*P = *0.005), DHA+RAP(3,3) (*P < *0.001), and DHA+AVA (3, 6) (*P < *0.001). Although the RMCBS score with DHA+RAP+AVA(3,3,3) was slightly higher than that with DHA+AVA(3,3), there was no significant difference between them (Fig. 1B). In supplemental monotherapy, the RMCBS scores showed a decreasing trend in all groups of mice (Fig. S1B). There was little change in RMCBS scores in each group of mice during the first 3 days p.i. From day 6 p.i., the RMCBS scores of the mice began to decrease, indicating that symptoms of ECM had already occurred. On day 9 p.i., the untreated mice had the lowest score and the treated mice had a higher score than the untreated mice. The neurological status of RAP (0) mice was better than that of the other treated groups and was significantly different from that of the untreated group (*P = *0.001). The scores of the AVA (3) and RAP (3) mice were also significantly different from that of the untreated group (*P = *0.004 and *P = *0.046, respectively). However, the AVA (6) group was not significantly different from the untreated group. The scores of mice in the RAP (0) group were not significantly different from those in the RAP (3) group and were not statistically different. The RMCBS scores of mice in the AVA (3) group were higher than those of mice in the AVA (6) group and were significantly different (*P = *0.054). There was no significant difference in groups with either RAP or AVA treatment modality compared with the untreated group on day 11 p.i. (Fig. S1B).

### Effect on the levels of parasitemia and survival rates in ECM.

In combination therapy, peripheral parasitemia measurements revealed increasing levels of parasitemia in all groups during the first 3 days of infection (Fig. 1C). After starting the combination treatment, parasitemia began to decrease in all groups. On day 8 p.i., there was no significant difference between the DHA+RAP (3, 0) and DHA+RAP(3,3) groups. Parasitemia was significantly lower in the DHA+AVA(3,3) than in the DHA+AVA (3, 6) treatment group (*P < *0.001). In the groups treated with DHA combined with RAP and AVA, the DHA+RAP+AVA(3,3,3) group had the minimum parasitemia, which was notably lower than the levels in the DHA+RAP+AVA (3, 0, 6) (*P < *0.001) and DHA+RAP+AVA(3,3,6) (*P < *0.001) groups. Nevertheless, there was no significant difference between DHA+RAP+AVA(3,3,3) and DHA+RAP+AVA (3, 0, 3). For the triple-drug combination compared to the double-drug combination, the parasitemia with DHA+RAP+AVA(3,3,3) was significantly lower than those with DHA+RAP (3, 0) (*P < *0.001), DHA+RAP(3,3) (*P = *0.001), and DHA + AVA (3, 6) (*P < *0.001) but was not different from that with DHA+AVA(3,3). The DHA+RAP+AVA (3, 0, 3) combination was only significantly different from DHA+RAP (3, 0) (*P < *0.001) versus DHA+AVA (3, 6) (*P = *0.001). Consequently, DHA+RAP+AVA(3,3,3) could be considered a candidate therapeutic combination with the three drugs (Fig. 1C). In supplemental monotherapy, peripheral parasitemia measurements revealed increasing levels of parasitemia in all groups during the first 3 days of infection (Fig. S1C). The level of parasitemia continued to increase exponentially after drug treatment in the medication group, showing no tendency to reduce parasitemia. The RAP (0) group was significantly different from the untreated group (*P = *0.012), but RAP (0) could not reduce parasitemia levels to a greater extent. There was no significant difference between the RAP (3) and untreated groups. The AVA (3) group was statistically different from the untreated group (*P = *0.043), but the difference was small and AVA (3) was not sufficient to reduce the effect of high parasitemia on ECM mice. There was no statistical difference between the AVA (6) and untreated groups. Therefore, monotherapy does not reduce the level of peripheral parasitemia (Fig. S1C).

In combination therapy, the survival rate of mice in the DHA+RAP+AVA(3,3,3) group was the highest among all the groups (Fig. 1D). All untreated mice died and exhibited specific ECM signs before day 10 p.i. None of the mice in any of the treated groups died due to ECM before 18 days. In the double-drug treatment, there was no significant difference in survival between the DHA+RAP (3, 0) and DHA+RAP(3,3) groups. Two modes of administration, DHA+AVA(3,3) and DHA+AVA (3, 6), differed greatly in their effects on mouse survival (*P = *0.0031), with DHA+AVA(3,3) yielding a higher percent survival. In DHA combined with RAP and AVA, there were significant differences among the four modes of regimens. The survival rates with DHA+RAP+AVA(3,3,3) and DHA+RAP+AVA (3, 0, 3) were higher than those with DHA+RAP+AVA (3, 0, 6) and DHA+RAP+AVA (3,3,6), and the treatment effect of DHA+RAP+AVA(3,3,3) was better than that of DHA+RAP+AVA (3, 0, 3) (*P = *0.0047). In summary, DHA+RAP (3, 0), DHA+RAP(3,3), and DHA+AVA(3,3) were screened out, and they had a better therapeutic effect than DHA. Among the triple-drug combinations, DHA+RAP+AVA(3,3,3) treatment was identified, which had a significantly higher survival rate than DHA+RAP (3, 0) (*P = *0.0031), DHA+RAP(3,3) (*P = *0.0048), and DHA+AVA(3,3) (*P = *0.0084) (Fig. 1D). In supplemental monotherapy, all untreated mice began to die on day 8 p.i. with specific ECM signs, and the survival rate dropped to 0 at day 14 p.i. (Fig. S1D). The mice in the RAP (0), RAP (3), AVA (3), and AVA (6) groups began to die on day 9, day 9, day 6, and day 7 p.i., respectively, and all died within 18 days. The RAP (0) and RAP (3) groups were statistically different from the untreated group (*P = *0.011 and *P = *0.046). Although RAP can improve the survival rate of mice, both groups also died within 18 days p.i. and RAP did not extend the survival time of mice. The survival rates of AVA (3) and AVA (6) mice were not statistically significant compared with those of untreated mice. The results showed that treatment with RAP or AVA alone had a limited effect (Fig. S1D).

### Tissue-protective effects on the brain.

**FIG 2:**
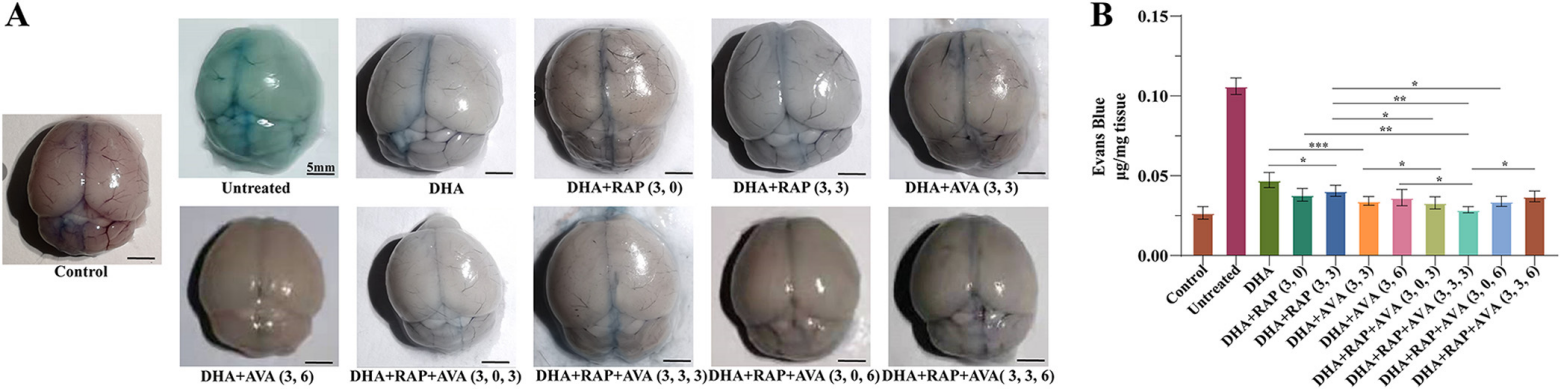
Assessment of BBB permeability. (A) Representative images of brain tissue following various treatments; (B) evaluation of vascular leakage by the Evans blue test. The statistical analysis method was one-way ANOVA and the LSD posttest was used for each one-way ANOVA. The error bars indicate standard deviation. Scale bars: 5 mm. *, *P < *0.05; **, *P < *0.01; ***, *P < *0.001.

In combination therapy, both visual inspection and Evans blue (EB) quantification indicated obvious EB leakage into the brain in untreated mice, slight EB leakage in the DHA-treated mice, and no significant EB leakage in the other treated groups, with the smallest amount of EB leakage in DHA+RAP+AVA (3, 3, 3) group (Fig. 2A and B). The DHA was statistically different from all other groups. In DHA monotherapy compared to DHA combined with RAP or RAP, the difference of vascular leakage between DHA and DHA+RAP(3,3) was the smallest (*P = *0.043). The difference of vascular leakage between DHA and DHA+AVA(3,3) was the largest (*P < *0.001). In the therapy with DHA combined with RAP or AVA, there was no significant difference in EB leakage between DHA+RAP (3, 0) and DHA+RAP(3,3). In the triple-drug treatment, there was only a significant difference between DHA+RAP+AVA(3,3,3) and DHA+RAP+AVA(3,3,6) (*P = *0.016), and there was no significant difference between the other groups. For the combination of three drugs compared to double-drug combination, vascular leakage was significantly different between the DHA+RAP+AVA (3, 0, 3) and DHA+RAP(3,3) groups (*P = *0.028). There was significant difference in EB leakage between the DHA+RAP+AVA(3,3,3) and DHA+RAP (3, 0) (*P = *0.008), DHA+RAP(3,3) (*P = *0.001), and DHA+AVA(3,3) (*P = *0.027) groups, but the DHA+RAP+AVA(3,3,3) group was not significantly different from the DHA+AVA(3,3) group. In addition, the DHA+RAP+AVA (3, 0, 6) group was also statistically different from the DHA+RAP(3,3) group (*P = *0.049) (Fig. 2A and B). In supplemental monotherapy, both visual inspection and EB quantification indicated EB leakage into the brain in all groups of mice (Fig. S2A and B). Based on the quantitative analysis, it is clear that the untreated group had the most EB leakage and the RAP (0) group had the lowest leakage. The amount of EB leakage in the RAP (0) and RAP (3) groups was higher than that in the AVA (3) and AVA (6) groups. The RAP (0) group had a significant difference from the untreated group (*P = *0.003). Meanwhile, the RAP (3) group was also significantly different from the untreated group (*P = *0.021). There was no significant difference in EB leakage between the AVA (3), AVA (6), and untreated groups. Treatment with RAP on day 0 p.i. had a greater effect on blood-brain barrier (BBB) permeability than treatment with RAP on day 3 p.i., but there was no statistically significant difference between the two treatments. There was also no statistically significant difference between the two treatments regardless of whether AVA was administered to the ECM on day 3 p.i. or on day 6 p.i. (Fig. S2A and B).

**FIG 3:**
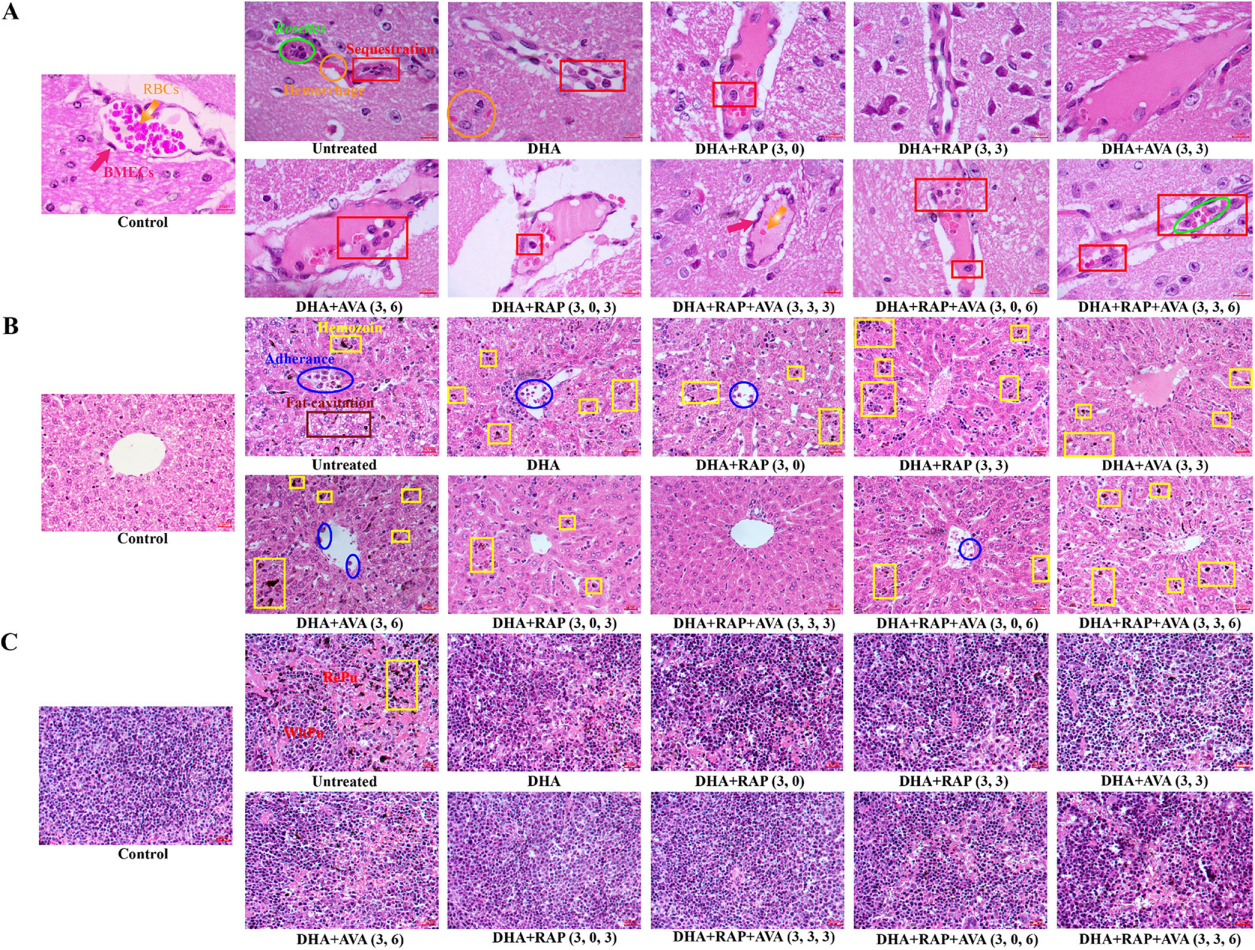
Histopathology staining of the brain, liver, and spleen. (A) Hematoxylin-eosin (HE) staining of the brain (magnification, ×1,000). The yellow arrow points to red blood cells (RBCs). The red arrow points to brain microvascular endothelial cells (BMECs). Scale bars: 10 μm. (B) HE staining of the liver (magnification, ×400). Scale bars: 20 μm. (C) HE staining of the spleen (magnification, ×400). The red areas represent RePu, and the blue areas represent white pulp (WhPu). Scale bars: 20 μm.

In combination therapy, the adhesion and accumulation of leukocytes in cerebral vessels are correlated with brain inflammation, which is a key feature of ECM (24). DHA+RAP+AVA(3,3,3) effectively reduced the aggregation of inflammatory cells and decreased the sequestration of iRBCs in the brain capillaries in all groups (Fig. 3A). Histological lesions did not appear in the control. Untreated mice displayed prominent aggregation of iRBCs and leukocytes in the lumen blood vessels, which accumulated together to form a rosette effect. The DHA+RAP (3, 0) group showed a few leukocytes obstructing the vascular lumen and a few scattered hemorrhagic spots around the vessels. The DHA+RAP(3,3) group presented less vascular obstruction and leukocyte aggregation. The histopathological changes in the brain were more different between the DHA+AVA(3,3) and DHA+AVA (3, 6) groups. Few iRBCs and little inflammatory cells were found in the brain capillaries of DHA+AVA(3,3)-treated mice, whereas there was more leukocyte deposition in the lumen of DHA+AVA (3, 6)-treated mice. In the triple-drug treatment group, capillaries in DHA+RAP+AVA(3,3,3)-treated mice were almost free of iRBCs and little inflammatory cells adhesion and recruitment, followed by the DHA+RAP+AVA (3, 0, 3) group, and there was little leukocyte aggregation in cerebral vessels. In contrast, the DHA+RAP+AVA (3, 0, 6) and DHA+RAP+AVA(3,3,6) groups still had a large number of inflammatory cells and iRBCs in the cerebrovasculature. In the combined treatment of the two drugs, both the DHA+RAP(3,3) and DHA+AVA(3,3) groups were screened, and DHA+AVA(3,3) was more protective in brain tissue. For the triple-drug combination, intracerebral vascular obstruction and hemorrhage were less frequent with DHA+RAP+AVA(3,3,3) than with the double-drug combination. This finding indicated that DHA+RAP+AVA(3,3,3) yielded a prominent improvement in cerebral vascularity and had a significant protective effect against ECM-induced brain injury (Fig. 3A). In the study of supplemental monotherapy, untreated mice showed significant accumulation of iRBCs and leukocytes in cerebral vessels, which accumulated together to form a rosette effect, with scattered hemorrhagic spots visible around the blood vessels (Fig. S3A). The iRBCs and inflammatory cell infiltration were reduced in the RAP (0) and RAP (3) groups compared with those in the untreated group, but aggregates were still present. There were a large number of iRBCs and inflammatory cell adhesion and recruitment in the AVA (3) and AVA (6) groups. Compared with the untreated group, the RAP (0) and RAP (3) showed a protective effect on brain tissue, but the therapeutic effect was limited to achieve the optimal therapeutic effect. AVA (3) and AVA (6) displayed poor protection against ECM-induced brain injury, and there were still more iRBCs and leukocyte aggregation (Fig. S3A).

### Tissue-protective effects on the liver.

In combination therapy, histological analysis of uninfected mice was used as a control. Among all treatment groups, the DHA+RAP+AVA(3,3,3) group had the most structurally intact hepatic lobules and exhibited the greatest protective capacity for the liver (Fig. 3B). The hepatic lobule structure in the untreated group exhibited severe damage, and the hepatocyte cytoplasm was darkly stained because of a lack of glycogen. The liver contained a large number of fat vacuoles. A large number of leukocytes were sequestered in the venous vessels. In the DHA+RAP (3, 0) group, the hepatic plates were not closely arranged, the number of leukocytes blocked the venous vessels, and hemozoin deposition was reduced. DHA+RAP(3,3) had a stronger protective effect on the structure of the hepatic lobules than did DHA+RAP (3, 0), with a tighter arrangement between hepatocytes and a significant reduction in leukocyte recruitment. The DHA+AVA(3,3) combination restored the normal structure of the hepatic lobules, but there was still a small amount of hemozoin deposition. The hepatocytes of the DHA+AVA (3, 6) group were disorganized and not arranged radially, with darkly stained cytoplasm and massive deposits of hemozoin. For the triple-drug treatment mice, the hepatocytes of the DHA+RAP+AVA(3,3,3) group were arranged in a tight radial pattern around the central vein with clear distinct boundaries. The hepatocyte cytoplasm was rich in lightly stained due to the normal nutritional status of the body and formed a pinnate appearance. There was scarcely any adhesion or aggregation of iRBCs or leukocytes in the venous vessels. The hepatic lobule of DHA+RAP+AVA (3, 0, 3) mice was normal in structure, with tightly arranged hepatocytes in a radial pattern, light cytoplasm staining of hepatocytes, and only a tiny amount of malarial hemozoin deposition and sequestration of few leukocytes in the central vein. In the DHA+RAP+AVA (3, 0, 6) and DHA+RAP+AVA(3,3,6) groups, the hepatic lobules were disrupted, and hepatocytes were irregularly arranged, with more hemozoin deposition. In the double-drug combination treatment, the DHA+RAP(3,3) and DHA+AVA(3,3) had better liver protection ability. The DHA+RAP+AVA (3, 0, 3) and DHA+RAP+AVA(3,3,3) combinations were screened in the triple-drug combination remedy, and these treatment groups had essentially normal hepatic lobular architecture, significant improvement in nutritional status, and a notable reduction in hemozoin compared with the double-drug treatment group (Fig. 3B). In supplemental monotherapy, the untreated group had severe structural damage to hepatic lobules and dark staining of the hepatocyte cytoplasm because of a lack of glycogen (Fig. S3B). A large number of leukocytes and iRBCs were retained in the vessels and blocked the venous vessels, and a large amount of hemozoin was deposited. The RAP (0) and RAP (3) groups had disorganized hepatocytes and still more deposition of hemozoin, but iRBCs and leukocyte adhesion and aggregation were reduced compared with those of the untreated group. The AVA (3) and AVA (6) groups showed destruction of hepatic lobules and irregular arrangement of hepatocytes, with more hemozoin deposition. Many iRBCs and leukocyte adhesion and aggregation were similar to those in the untreated group, which meant that these groups did not show a significant improvement. Although RAP treatment reduced cell aggregation in the liver, it did not significantly improve the damage to liver tissue. The liver tissue structure of mice treated with AVA alone was similar to that of the untreated group and showed no obvious protective effect on liver tissue (Fig. S3B).

In the next combination therapy, we quantified hemozoin deposition in liver tissue sections stained with hematoxylin-eosin (HE) (Fig. 1E). The ability of DHA combined with RAP or AVA to clear hemozoin is better than that of DHA monotherapy, and the ability of DHA combined with RAP and AVA to metabolize hemozoin is stronger than that of DHA combined with RAP or AVA. Compared with DHA monotherapy, significant differences were observed for DHA+RAP(3,3) (*P = *0.025) and DHA+AVA(3,3) (*P < *0.001). Therefore, DHA+RAP(3,3) and DHA+AVA(3,3) serve as candidate therapies for combination therapy with the two drugs. For DHA combined with RAP and AVA, the DHA+RAP+AVA(3,3,3) group had the lowest hemozoin content. The hemozoin area of the DHA+RAP+AVA(3,3,3) group was significantly smaller than those of the DHA+RAP (3, 0) (*P < *0.001) and DHA+AVA(3,3) (*P = *0.032) groups. There was a significant difference between DHA+RAP+AVA (3, 0, 3) and DHA+RAP(3,3) (*P = *0.017) but not DHA+AVA(3,3) (Fig. 1E). Therefore, DHA+RAP+AVA(3,3,3) was selected as a candidate remedy for the triple-drug combination. In supplemental monotherapy, quantitative analysis of hemozoin deposition in liver tissue was performed (Fig. S1E). Hemozoin deposition was highest in the untreated group. The RAP (0) and RAP (3) groups had less hemozoin deposition. Hemozoin deposition in the AVA (3) and AVA (6) groups was similar to that in the untreated group. However, there was no significant difference in liver hemozoin deposition between the treated group and the untreated group (Fig. S1E).

### Protective effects on the spleen tissue.

In combination therapy, histological analysis of the spleen demonstrated a significantly widened red pulp (RePu) region and atrophy in the white pulp (WhPu) area in the untreated group and the DHA, DHA+AVA (3, 6), and DHA+RAP+AVA(3,3,6) groups, indicating severe anemia and more extramedullary hematopoiesis (Fig. 3C). The RePu area in the DHA+RAP (3, 0), DHA+RAP(3,3), DHA+AVA(3,3), and DHA+RAP+AVA (3, 0, 6) groups was smaller than in the above-mentioned four groups, indicating improvement of anemia. The RePu region of the DHA+RAP+AVA (3, 0, 3) and DHA+RAP+AVA(3,3,3) groups was smaller than those of the other eight treatment groups, and the proportion of WhPu was larger. The spleen structures of the DHA+RAP+AVA (3, 3, 3) and DHA+RAP+AVA (3, 3, 3) groups were similar to those of the control group. There was no significant difference between the DHA+RAP(3,3) and DHA+RAP (3, 0) groups in the RePu region. The RePu width of the DHA+AVA(3,3) group was smaller than that of the DHA+AVA (3, 6) group. Compared with that with DHA monotherapy, the RePu region with DHA+RAP and DHA+AVA was significantly reduced, indicating a decrease in extramedullary hematopoiesis. Therefore, the therapeutic effect of the combination with both drugs on the spleen was better than that of DHA monotherapy. In DHA combined with both RAP and AVA, DHA+RAP+AVA (3, 0, 3) and DHA+RAP+AVA(3,3,3) yielded the smallest RePu area, indicating minimal extramedullary hematopoiesis. Moreover, DHA+RAP+AVA (3, 0, 3) and DHA+RAP+AVA(3,3,3) superior to DHA in combination with RAP or AVA for the spleen (Fig. 3C). In supplemental monotherapy, histological analysis of the spleen showed significant widening of the RePu area and atrophy of the WhPu area in the untreated group and the RAP (0), RAP (3), AVA (3), and AVA (6) groups, indicating severe anemia and more extramedullary hematopoiesis (Fig. S3C). The spleen structure in the treated group was similar to that in the untreated group, with no significant improvement (Fig. S3C).

In combination therapy, a quantitative analysis of the RePu area in the spleen was performed (Fig. 1F). In both drug combination treatments, the RePu region was smaller in the DHA+RAP(3,3) group than in the DHA+RAP (3, 0) group. The RePu area of the DHA+AVA(3,3) group was smaller than that of the DHA+AVA (3, 6) group. For the double-drug combination compared to the single-drug treatment, the RePu areas of the DHA+RAP(3,3) (*P = *0.004) and DHA+AVA(3,3) (*P = *0.001) groups were significantly different from that of the DHA group. Among the triple-drug combination therapy groups, the DHA+RAP+AVA(3,3,3) group had the smallest RePu area. There was no significant difference between DHA+RAP+AVA (3) and DHA+RAP+AVA (3, 0, 3). In contrast, the DHA+RAP+AVA (3, 0, 6) and DHA+RAP+AVA(3,3,6) groups had a larger RePu area and poorer treatment outcomes. For the double-drug combination treatment, there were significant differences in DHA+RAP+AVA (3, 0, 3) and DHA+RAP+AVA(3,3,3) compared with DHA+RAP(3,3) and DHA+AVA(3,3) (*P < *0.001). However, DHA+RAP+AVA (3, 0, 6) and DHA+RAP+AVA(3,3,6) were not significantly different from DHA+RAP and DHA+AVA, and their protective effects on the spleen were not improved. These results demonstrated that DHA+RAP(3,3) and DHA+AVA(3,3) were more effective than DHA in both drug combination treatments. The therapeutic effects of DHA+RAP+AVA (3, 0, 3) and DHA+RAP+AVA(3,3,6) were superior to those of DHA+RAP and DHA+AVA (Fig. 1F). In supplemental monotherapy, we performed quantitative analysis of hemozoin deposition in spleen tissue. There was no statistical difference in the RAP (0), RAP (3), AVA (3), and AVA (6) groups from the untreated group (Fig. S1E). The results showed that monotherapy was unable to reduce hemozoin compared to that in the untreated group.

In combination therapy, quantitative analysis of hemozoin deposition in spleen tissue showed that the DHA+RAP(3,3) and DHA+AVA(3,3) combinations yielded lower hemozoin contents (Fig. 1E). There was no significant difference between DHA+RAP(3,3) and DHA, and there was a significant difference between DHA+AVA(3,3) and DHA (*P = *0.012). In DHA combined with RAP and AVA, the smallest amount of hemozoin was found with DHA+RAP+AVA(3,3,3), but the amount did not differ significantly from that with either DHA+RAP(3,3) or DHA+AVA(3,3) (Fig. 1E). In supplemental monotherapy, a quantitative analysis of the RePu area in the spleen was performed (Fig. S1F). The untreated group had the largest RePu region. Compared with that of the untreated group, RAP (0) reduced the RePu area (*P = *0.039). There was no significant difference between the RAP (3), AVA (3), and AVA (6) groups and untreated groups. Although RAP (0) reduced the RePu area, the RePu was still high and anemia still existed (Fig. S1F). The results demonstrated that monotherapy could not reduce the RePu area and did not improve anemia symptoms.

## DISCUSSION

CM is a fatal complication caused by P. falciparum malaria. While the use of ART has tremendously enhanced the clinical outcome of malaria, the high mortality and disability rates associated with severe malaria remain a severe clinical problem. The most fatal pathological damage of CM is neurological lesions, and antimalarial drugs can only reduce the level of parasitemia in peripheral blood and cannot prevent or repair the brain microvascular injury that occurs. Even those who survive will still have lifelong neurological sequelae, including cognitive impairment, language impairment, and motor impairment. In this context, the development of combination therapies is expected to inhibit disease progression from all aspects of pathogenesis and provide new therapeutic ideas and directions for the treatment of CM.

In the early stage of adjuvant therapy, experiments were established for single-drug treatments and results for body weight, RMCBS score, parasitemia, survival rates, BBB permeability, and histopathology were compared (Fig. S1 to S3). The preventive effect of monotherapy on ECM compared with the untreated group appeared to be limited, although some of the indicators of rapamycin were different from those in the untreated group, and the effect was poor on the basis of no additional antimalarial treatment. According to the pathogenesis of ECM, the appearance of iRBCs in brain capillaries is the initial stage of ECM progression, so antimalarial treatment that first eradicates *Plasmodium* is the key stage of ECM treatment. It has been reported that in a mouse model of ECM, the mTOR inhibitor RAP used within the first 4 days of infection prevented ECM but had no significant effect on the level of peripheral parasitemia (25). This is consistent with the results of our experiments in which treatment of mice with RAP either on day 0 p.i. or on day 3 p.i. reduced the permeability of the BBB and prolonged the survival rate compared to those in the untreated group, but the therapeutic effect of RAP alone is limited compared to RAP combined with antimalarials because of the inability to reduce parasitemia levels. Therefore, these results suggest that RAP could be a powerful candidate to open up new avenues of adjuvant therapy for CM. Therefore, in order to achieve a better therapeutic effect, this experiment added adjuvant RAP therapy. AVA acts as a neuroprotective agent, but reports suggest that AVA alone does not prevent death from ECM or reduce parasitemia in infected mice (20). This was not significantly different from the findings for mice treated with AVA alone compared to the untreated group in our experiment: AVA prolonged survival but survival was not statistically different. In addition, it could not reduce the permeability of the BBB or reduce the damage to tissue cells. Although studies have shown that AVA alone is not effective, combined antimalarial agents significantly delayed death in mice and reduced ECM symptoms (21, 22). Therefore, AVA can also be used as an adjuvant drug to enhance the optimal effect of ECM treatment.

The effectiveness of RAP as an immunosuppressant is likely due to its ability to inhibit effector T-cell differentiation and suppress effector T-cell metabolism and function (26). However, only the use of DHA combined with RAP does not directly exert neuroprotective effects, so blocking apoptosis of endothelial cells (ECs) and neuronal cells from the terminal link is also necessary. In this investigation, this experiment combined an additional neuroprotective drug, AVA, based on the DHA and RAP study. AVA is active against P. falciparum (12) and is considered to be an outstanding adjuvant in the treatment of CM. It has been shown that AVA inhibits caspase-mediated apoptosis of endothelial and glial cells and systemic and cerebral inflammatory responses by reducing the plasma and cerebrospinal fluid levels of an apoptotic factor, CXCL10 (27). CXCL10 is pathologically involved in CM (28–31) and has also been reported to play a role in central nervous system (CNS) neuronal injury, inhibition of angiogenesis, and interference with vascular endothelial growth factor (VEGF) function. In this study, DHA in combination with RAP and AVA was used to treat the ECM mouse model, and different medication modes were used to observe the additive protective effects on mice, as well as to compare the effects of the triple-drug combination.

DHA combined with RAP, administered on either day 0 or day 3 p.i., protected mice from ECM neuropathology and death. This is consistent with the results reported in the literature that treatment with RAP until day 4 p.i. protected mice from ECM. The DHA+RAP (3, 0) and DHA+RAP(3,3) groups did not present statistically significant differences in body weight, RMCBS score, parasitemia, or survival rate. In terms of histopathology, the DHA+RAP(3,3) group showed slightly less leukocyte aggregation than the DHA+RAP (3, 0) group and slightly reduced hemozoin and RePu areas, but overall, the difference in effect between the two treatments was not significant. However, compared with that of the DHA group, the survival in both groups was extended to 21 days, and the pathological damage to the brain, liver, and spleen was reduced. In the combination of DHA and AVA, there was a great difference between the results of using AVA on day 3 and day 6 p.i. DHA+AVA(3,3) significantly inhibited weight loss, improved RMCBS scores, reduced parasitemia, and improved the survival rate compared with those with DHA+AVA (3, 6). Meanwhile, DHA+AVA(3,3) was more prominent than DHA+AVA (3, 6) in improving histopathological damage. Compared with DHA, DHA+AVA(3,3) significantly improved the RMCBS scores, reduced parasitemia, prolonged survival, and showed an enhanced ability to protect tissue.

Among the triple-drug combinations, DHA+RAP+AVA(3,3,3) and DHA+RAP+AVA (3, 0, 3) had additive therapeutic effects on ECM, especially DHA+RAP+AVA(3,3,3), which showed the best therapeutic effect. In contrast, DHA+RAP+AVA (3, 0, 6) and DHA+RAP+AVA(3,3,6) did not show additive therapeutic effects compared with the two-drug combination. DHA+RAP+AVA(3,3,3) and DHA+RAP+AVA (3, 0, 3) significantly prolonged the survival rate of mice, reduced parasitemia, improved the RMCBS score, and improved the body weight of mice compared with the double-drug combination. DHA+RAP+AVA(3,3,3) reached an even 100% survival rate in mice as of D22 p.i. DHA+RAP+AVA (3, 0, 6) did not differ much from DHA+RAP and DHA+AVA(3,3) terms of basal indicators. DHA+RAP+AVA(3,3,6) had a worse treatment effect than DHA in terms of body weight, RMCBS score, parasitemia, and survival rate, which are the underlying indicators and did not show additive effects of DHA. In terms of BBB permeability, the EB in the DHA+RAP+AVA(3,3,3) group showed the least amount of leakage, indicating that this combination was the most protective of brain tissue. The second best combinations in terms of BBB permeability were DHA+RAP+AVA (3, 0, 3) and DHA+AVA(3,3). There was little difference in the exudation of EB between them, and they had a strong protective ability in brain tissue. In terms of histopathology, DHA+RAP+AVA (3, 0, 3) and DHA+RAP+AVA(3,3,3) showed significant advantages in the protection of the brain, liver, and spleen. In contrast to the combinations of two drugs, there was little leukocyte recruitment in the cerebral vessels and scattered hemorrhage in the cerebral parenchyma. The liver tissue maintained a relatively intact hepatic lobule structure with little hemozoin deposition. The area of RePu in the spleen was observably reduced, indicating a significant reduction in extramedullary hematopoiesis. The protective effects of DHA+RAP+AVA (3, 0, 6) and DHA+RAP+AVA(3,3,6) on histopathology were similar to those of the combination of two drugs and did not show obvious advantages.

DHA combined with RAP and AVA has been identified to have additive antimalarial effects, improving neurological signs, pathological outcomes, and behavioral manifestations in mice with ECM, but further studies are needed to determine its specific mechanism of action. Next, we will further study the molecular protective mechanisms associated with the triple-drug combination through multiomics platforms such as transcriptomics and metabolomics and spatial metabolomics combined with the gut microbiota (32, 33). Subsequent studies of differential metabolites and metabolic pathways in triple-drug combinations, as well as exploration of the signal transduction pathways associated with drug combinations, will provide more evidence and new targets for improving the clinical treatment of human CM.

In conclusion, DHA in combination with RAP and AVA has additive therapeutic effects in treating ECM. It significantly improved ECM symptoms, reduced microvascular blockages in brain tissue, and markedly improved CM neurological impairment. Among the triple-drug combinations, the DHA+RAP+AVA(3,3,3) combination was identified as the optimal treatment. It offers a potential treatment strategy for CM and will play an important role in reducing the disease burden of CM.

## MATERIALS AND METHODS

### Ethics statement.

The animals were maintained and used according to the Regulations for the Administration of Affairs Connecting Experimental Animals in China and the international research animal use guidelines. All efforts were made to minimize animal suffering. The protocol was approved by the Institutional Animal Care and Use Committee of the Hubei University of Medicine (HBMU-S20160414), and the mice were housed in the Hubei Medical College Laboratory Animal Center.

### Mice and infection with Plasmodium berghei ANKA (PbA).

Female C57BL/6 mice (weight, 18 to 22 g; age, 8 to 10 weeks) were purchased from the company HNSJA Co., Ltd. (Changsha, China), and maintained under specific-pathogen-free conditions. The mice were fed a UV-illuminated diet and pure water, which maintained the appropriate standards of living and feeding experimental conditions (25 ± 3°C). All mice were acclimatized to the housing for 1 week before the study. The animal experiments were repeated three times, and the numbers for each group were 8, 10, and 10 in different batches, respectively.

The Plasmodium berghei ANKA strain was removed from liquid nitrogen and thawed rapidly in a 37°C water bath. The parasites were maintained by successive infection of C57BL/6 mice. Infections were initiated in C57BL/6 mice via intraperitoneal injection of 1 × 10^6^ PbA-infected red blood cells (iRBCs).

### Experimental grouping and drug treatment scheme.

**FIG 4:**
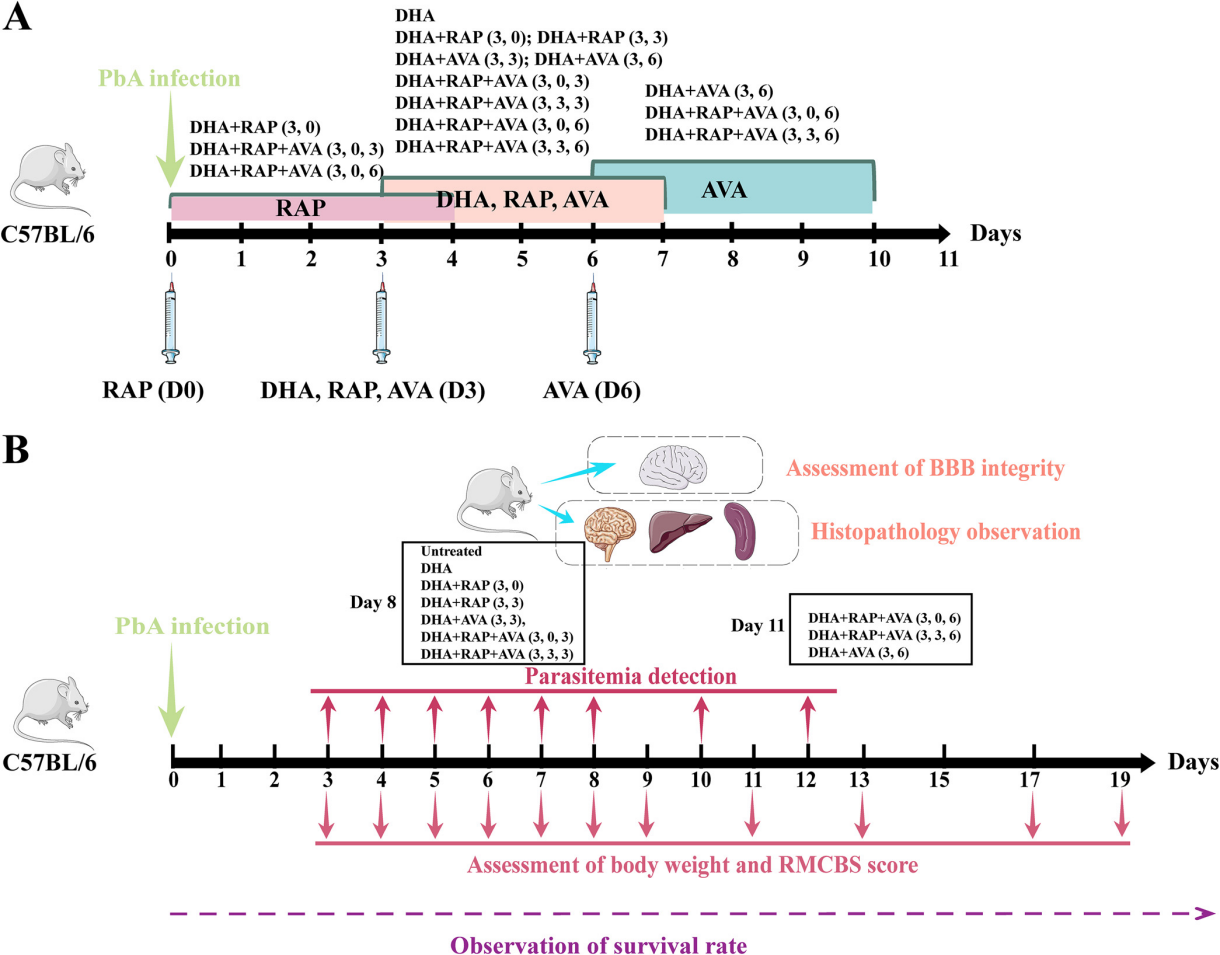
Experimental design. (A) Experimental grouping and therapy scheme. The experimental cerebral malaria (ECM) model was established by infecting C57BL/6 mice with Plasmodium berghei ANKA (PbA) on day 0 (D0) postinfection (p.i.). Therapy with dihydroartemisinin (DHA), rapamycin (RAP), and atorvastatin (AVA) was administered on day 0, day 3, and day 6 p.i., with the respective administration times for each group shown. The day of infection was recorded as day 0 p.i. (B) Detection of pathogenesis indicators. Mice were monitored for body weight, rapid murine coma and behavioral scale (RMCBS) score, parasitemia, survival rate, blood-brain barrier (BBB) integrity, and histopathology. Boxes for day 8 and day 11 p.i. indicate which groups were subjected to histopathological observation and BBB integrity assessment.

According to different medication strategies, mice were randomly divided into 10 groups, as shown in Fig. 4. DHA, RAP, and AVA were dissolved in 5% dimethyl sulfoxide (DMSO) in 0.9% NaCl at 3 mg/kg, 5 mg/kg, and 40 mg/kg of body weight, respectively. The untreated mice were injected with 5% DMSO on day 3 postinfection (p.i.) as a control. The DHA was treated with 3 mg/kg DHA on day 3 p.i. The DHA+RAP (3, 0) group started treatment with 5 mg/kg RAP and 3 mg/kg DHA on day 0 and day 3 p.i., respectively. The DHA+RAP(3,3) group was treated with 3 mg/kg DHA and 5 mg/kg RAP on day 3 p.i. at the same time. The DHA+AVA(3,3) group began therapy with 3 mg/kg DHA and 40 mg/kg AVA on day 3 p.i., and the DHA+AVA (3, 6) group started treatment with 3 mg/kg DHA and 40 mg/kg AVA on day 3 and day 6 p.i., respectively. The DHA+RAP+AVA (3, 0, 3) group began treatment with 5 mg/kg RAP, 3 mg/kg DHA, and 40 mg/kg AVA on day 0, day 3, and day 3 p.i., respectively. The DHA+RAP+AVA(3,3,3) group was treated with 3 mg/kg DHA, 5 mg/kg RAP, and 40 mg/kg AVA simultaneously on day 3 p.i. The DHA+RAP+AVA (3, 0, 6) group started treatment with 5 mg/kg RAP, 3 mg/kg DHA, and 40 mg/kg AVA on day 0, day 3, and day 6 p.i., respectively. The DHA+RAP+AVA(3,3,6) group began therapy with 3 mg/kg DHA, 5 mg/kg RAP, and 40 mg/kg AVA on day 3, day 3, and day 6 p.i. Each drug was administered for 5 consecutive days. There was a crossover in the course of administration (Fig. 1). In addition, this study subsequently complemented an experiment of monotherapy for ECM. The experiment contained 5 groups (11 mice for each group): untreated infection group, rapamycin treatment group on day 0 p.i. [RAP (0)], rapamycin treatment group on day 3 p.i. [RAP (3)], atorvastatin treatment group on day 3 p.i. [AVA (3)], and atorvastatin treatment group on day 6 p.i. [AVA (6)]. According to different treatment strategies, untreated mice were injected with 5% DMSO on day 3 p.i. as a control. The RAP (0) group was treated with 5 mg/kg RAP on day 0 p.i., and the RAP (3) group started treatment with 5 mg/kg RAP on day 3 p.i. The AVA (3) group began therapy with 40 mg/kg AVA on day 3 p.i., and the AVA (6) group started treatment with 40 mg/kg AVA on day 6 p.i. Each drug was administered for 5 consecutive days.

### Detection of basic indicators.

The mice were monitored for daily body weight, neurological signs, parasitemia, and survival rate (Fig. 4). Neurological signs were evaluated using the rapid murine coma and behavioral scale (RMCBS) starting from day 0 p.i. (34). The score utilizes 10 parameters (gait, balance, motor performance, body position, limb strength, touch escape, pinna reflex, toe pinch, aggression, and grooming) and can be used to assess a subject mouse within 3 min. The RMCBS is scored from 0 to 20, with a 0 score correlating with the lowest function and a 20 score with the highest function. The mice were considered ECM positive if they gradually showed characteristics such as unstable walking, ataxia, ruffling with swaths of hair out of place, absence of body extension, decreased toe pinch and disappearance of pinna reflex, convulsions, coma symptoms, and even death. Parasitemia was assessed by Giemsa-stained thin blood smears using light microscopy at a magnification of ×1,000 (BX53; Olympus, Japan). Parasitemia was checked and quantified by counting the number of iRBCs in at least 1,000 RBCs (27).

### Assessment of BBB integrity.

The protective effect on the brain was assessed by the integrity of the blood-brain barrier (BBB) (35). Evans blue (EB; 1%) dye was diluted in 0.9% NaCl. Each mouse was injected with 200 μL 1% EB solution via the tail vein on day 8 and day 11 p.i. The dye was allowed to circulate for 30 min, and then mice were anesthetized and perfused with 0.9% NaCl through the heart to the right atrium for outflow of the clarifying fluid. Brains were isolated quickly, photographed, weighed, and then placed into a 1.5-mL centrifuge tube for grinding. Brains were incubated in 1 mL/sample formamide at 37°C for 48 h. These samples were centrifuged at 1,000 × *g* for 10 min, absorbance was measured by a microplate reader (Gene Company Limited, Synergy HT, USA) at 630 nm, and EB was quantified according to a standard curve (36). The results were expressed as microgram per milligram of EB tissue.

### Histopathology.

The brains, livers, and spleens were collected immediately after euthanasia and washed three times in cold phosphate-buffered saline (PBS) to remove blood. Then, these were immobilized with 4% paraformaldehyde for 24 h and embedded in paraffin. Serial 4-μm-thick horizontal sections were made, stained with hematoxylin-eosin (HE), and examined for microvascular obstruction and leakage. The area of accumulation of hemozoin was measured in the liver and spleen stained with HE, and the area of the red pulp (RePu) and white pulp (WhPu) in the spleen was determined using Olympus cellSens standard 1.13 software (Olympus, Japan). Five fields were randomly selected for each section (2). Sections were examined by light microscopy (Motic BA210), and images were taken using Olympus cellSens standard 1.13 software.

### Statistical analysis.

Data were analyzed using the Statistical Package for Social Sciences (SPSS Inc., USA). The results are expressed as means ± standard deviations (SD). The statistical analysis method was one-way analysis of variance (ANOVA) and the post hoc comparative analysis done after ANOVA, also known as the *t* test (LSD) posttest was used for each one-way ANOVA. Survival rate analysis was assessed by the log-rank (Mantel-Cox) test. A difference was considered significant for *P* values of <0.05. In figures, the symbols *, **, and *** represent *P* values of <0.05, <0.01, and <0.001, respectively. When the *P* value is less than 0.001, it is represented as *P < *0.001.
