# Supplementary material for: Additive Therapy of Plasmodium berghei-Induced Experimental Cerebral Malaria via Dihydroartemisinin Combined with Rapamycin and Atorvastatin
Source: Microbiol Spectr. 2023 Mar 22;11(2):e02317-22. doi: 10.1128/spectrum.02317-22 (PMC10101104; doi:10.1128/spectrum.02317-22)
Supplement: Supplemental file 1 — Supplemental material. Download spectrum.02317-22-s0001.pdf, PDF file, 1.1 MB [file spectrum.02317-22-s0001.pdf]

Additive therapy of *Plasmodium berghei* induced experimental cerebral malaria via dihydroartemisinin combined with rapamycin and atorvastatin.

Xiaonan Song*, Weijia Cheng*, Huiyin Zhu, Yuting Li, Jian Li#

School of Basic Medical Sciences, Hubei University of Medicine, Shiyan 442000, China

Running Title: Additive therapy of ECM via triple-drug of DHA+RAP+AVA

* These authors contributed equally

#Corresponding author
Jian Li, E-mail: yxlijian@163.com. Tel.: 86-719-8891141.

Supporting information

Supporting information 1: Supporting methods and results

Supporting information 2: Fig S1 Monitoring indicators associated with the ECM model receiving different treatments. (A) The bodyweight receiving different treatments. (B) The rapid murine coma and behavioral scale (RMCBS) score curve receiving different treatments. (C) The parasitemia receiving different treatments. (D) Survival rate curve analysis. (E) Analysis of the hemozoin proportion in the liver and spleen in the different treatment groups. (F) Quantification of the red pulp (RePu) area for the treatment groups. *, $p < 0.05$; **, $p < 0.01$; ***, $p < 0.001$. The statistical analysis method was one-way ANOVA and the LSD test was used as the post-test for one-way ANOVA analysis. Survival rate analysis was assessed by the log-rank (Mantel-Cox) test. The error bars were the standard deviation.

Supporting information 3: Fig S2 Assessment of BBB permeability. (A) Representative images of brain tissue following various treatments. (B) The Evans blue test was used to evaluate vascular leakage. Scale bars: 5 mm. *, $p < 0.05$; **, $p < 0.01$; ***, $p < 0.001$. The statistical analysis method was one-way ANOVA and the LSD test was used as the post-test for one-way ANOVA analysis. The error bars were the standard deviation.

Supporting information 4: Fig S3. Histopathology staining of the brain, liver, and spleen. (A) Hematoxylin-eosin (HE) staining of the brain (1000×). Scale bars: 20 μm. (B) HE staining of the liver (400×). Scale bars: 20 μm.

(C) HE staining of the spleen (400×). Scale bars: 20 μm.

Supporting methods and results

1. Materials and methods

This study complements an experiment of monotherapy for experimental cerebral malaria (ECM). The experiment was designed with 5 groups: untreated infection group (Untreated), rapamycin treatment group on Day 0 (D0) post-infection (p.i.) {RAP (0)}, rapamycin treatment group on D3 p.i. {RAP (3)}, atorvastatin treatment group on D3 p.i. {AVA (3)}, and atorvastatin treatment group on D6 p.i. {AVA (6)}. The number of mice in each group was 11. According to different treatment strategies, the untreated mice were injected with 5% DMSO on D3 p.i. as a control. The RAP (0) was treated with 5 mg/kg RAP on D0 p.i., the RAP (3) started treatment with 5 mg/kg RAP on D3 p.i.. The AVA (3) began therapy with 40 mg/kg AVA on D3 p.i., and the AVA (6) started treatment with 40 mg/kg AVA on D6 p.i.. Each drug was administered for 5 consecutive days. The mice were monitored for daily body weight, neurological signs, parasitemia, and survival rates. On D8 p.i., the untreated, RAP (0), RAP (3) and AVA (3) groups were subjected to histopathological staining of liver, spleen and brain tissues and assessment of blood-brain barrier (BBB) integrity. On D11 p.i., the AVA (6) group was performed to histopathological staining of liver, spleen and brain tissues and assessment of BBB integrity. The method of this detection index is completely consistent with the method in the text, and can refer to the methodology part of the text.

2.Results

1.1 Effect on body weight and neurological signs in ECM

The body weight of the mice in each group increased and then decreased after infection (Fig S1A). During the first two days of post infection (p.i.), the mice had not yet shown signs of disease, so their body weight increased slightly. On D3 p.i., the

mice tended to lose weight continuously because *Plasmodium falciparum*-infected red blood cells (iRBCs) began to increase in their bodies. On D9 p.i., the body weight of the drug-treated mice was higher than that of the untreated mice, but the mean body weight in the four groups drug-treated mice was not significantly different compared to the untreated group. Although the mean body weight was slightly higher in RAP (0) than the RAP (3) group, there was no significant difference between the two groups. The average body weight of AVA (3) was higher than that of AVA (6), but there was no statistical difference between the two groups. On D11 p.i., there was no significant difference in body weight between the untreated mice and the RAP or AVA treated mice (Fig S1A).

The rapid murine coma and behavioral scale (RMCBS) scores showed a decreasing trend in all groups of mice (Fig S1B). There was little change in RMCBS scores in each group of mice during the first 3 days p.i.. From the D6 p.i., the RMCBS scores of the mice began to decrease, indicating that symptoms of ECM had already occurred. On D9 p.i., the untreated mice had the lowest score and the treated mice had a higher score than the untreated mice. The neurological status of RAP (0) mice was better than that of the other treated groups and was significantly different from that of the untreated group (p = 0.001). The scores of AVA (3) and RAP (3) mice were also significantly different compared to untreated group (p = 0.004 and p = 0.046, respectively). However, the AVA (6) was not significantly different from untreated group. The scores of mice in RAP (0) were not significantly different from those in RAP (3) and were not statistically different. The RMCBS scores of mice in AVA (3) were higher than those of mice in the AVA (6) and were statistically different (p = 0.054). There was no significant difference in either RAP or AVA treatment modality compared with the untreated group on D11 p.i. (Fig S1B).

1.2 Effect on the level of parasitemia and survival rates in ECM

Peripheral parasitemia measurements revealed increasing levels of parasitemia in all groups during the first 3 days of infection (Figure S1D). The level of parasitemia continued to increase exponentially after drug treatment in the medication group, showing no tendency to reduce parasitemia. Compared with the untreated group, the

RAP (0) was significantly different from untreated group ($p = 0.012$), but could not reduce parasitemia levels to a greater extent. There was no significant difference between RAP (3) and untreated group. The AVA (3) was statistically different from untreated group ($p = 0.043$), but the difference was small and not sufficient to reduce the effect of high parasitemia on ECM mice. There was no statistical difference between AVA (6) and untreated group (Figure S1D). Therefore, monotherapy does not reduce the level of peripheral parasitemia.

All untreated mice began to die on D8 p.i. with specific ECM signs, and the survival rate dropped to 0 at day D14 p.i. (Figure S1D). The mice in RAP (0), RAP (3), AVA (3) and AVA (6) groups began to die on D9, D9, D6 and D7 p.i., respectively, and all died within 18 days. Compared with the untreated group, the RAP (0) and RAP (3) were statistically different from the untreated group ($p = 0.011$ and $p = 0.046$). Although RAP can improve the survival rate of mice, both groups also died within D18 p.i. and did not extend the survival time of mice longer. The survival rates of AVA (3) and AVA (6) mice were not statistically significant compared with those of untreated mice (Figure S1D). The results showed that treatment with RAP or AVA alone had a limited effect or poor treatment.

1.3 Tissue protective effects on the brain

Both visual inspection and Evans blue (EB) quantification indicated EB leakage into the brain in all groups of mice (Fig S2A and S2B). Based on the quantitative analysis, it is clear that the untreated group had the most EB leakage and RAP (0) had the lowest leakage. The amount of EB leakage in RAP (0) and RAP (3) groups was higher than that in AVA (3) and AVA (6) groups. The RAP (0) had a significant difference compared to untreated group ($p = 0.003$). Meanwhile, the RAP (3) was also significantly different from that of untreated group ($p = 0.021$). There was no significant difference in EB leakage between AVA (3), AVA (6) and untreated groups. Treatment with RAP on D0 p.i. had a greater effect on BBB permeability compared with treatment with RAP on D3 p.i., but there was no statistically significant difference between the two treatments. There was also no statistically significant difference between the two treatments regardless of whether AVA was administered to

the ECM on D3 p.i. or on D6 p.i. (Fig S2A and S2B).

Untreated mice showed significant accumulation of iRBCs and leukocytes in cerebral vessels, which accumulated together to form a rosette effect, with scattered hemorrhagic spots visible around the blood vessels (Fig S3A). The iRBCs and inflammatory cells infiltration were reduced in RAP (0) and RAP (3) groups compared with untreated groups, but aggregates were still present. There was a large number of iRBCs and inflammatory cells adhesion and recruitment in AVA (3) and AVA (6). Compared with untreated group, the RAP (0) and RAP (3) had a protective effect on brain tissue, but the therapeutic effect was limited to achieve the optimal therapeutic effect. The AVA (3) and AVA (6) showed poor protection against ECM-induced brain injury, and there were still more iRBCs and leukocyte aggregation (Fig S3A).

1.4 Tissue protective effects on the liver

The untreated group had severe structural damage to hepatic lobules and dark staining of the hepatocyte cytoplasm because of a lack of glycogen (Fig S3B). A large number of leukocytes and iRBCs were retained in the vessels and blocked the venous vessels, and a large amount of hemozoin was deposited. The RAP (0) and RAP (3) had disorganized hepatocytes and still more deposition of hemozoin, but iRBCs and leukocytes adhesion and aggregation were reduced compared with untreated group. The AVA (3) and AVA (6) groups showed destruction of hepatic lobules and irregular arrangement of hepatocytes with more hemozoin deposition. Compared with untreated group, the iRBCs and leukocytes adhesion and aggregation were similar, which did not show a significant improvement. Although RAP treatment reduced cell aggregation in the liver, it did not significantly improve the damage to liver tissue. The liver tissue structure treated with AVA alone was similar to that of the untreated group and showed no obvious protective effect on liver tissue (Fig S3B).

Quantitative analysis of hemozoin deposition in the liver tissue (Fig S1E). Hemozoin deposition was highest in the untreated group. The RAP (0) and RAP (3) had less hemozoin deposition. Hemozoin deposition in AVA (3) and AVA (6) groups was similar to that in untreated groups. However, there was no significant difference

in the liver hemozoin deposition between the treated group and the untreated group (Fig S1E).

1.5 Protective effects of the spleen tissue

Histological analysis of the spleen showed significant widening of the red pulp (RePu) area and atrophy of the WhPu area in untreated, RAP (0), RAP (3), AVA (3) and AVA (6) treated groups, indicating severe anemia and more extramedullary hematopoiesis. Compared with the untreated group, the spleen structure in the treated group was similar to that in the untreated group, with no significant improvement (Fig S3C).

Quantitative analysis of hemozoin deposition in the spleen tissue (Fig S1E). There was no statistical difference in RAP (0), RAP (3), AVA (3) and AVA (6) compared to untreated group (Fig S1E). The results showed that monotherapy was unable to reduce hemozoin compared to the untreated group.

A quantitative analysis of the RePu area in the spleen was performed (Fig S1F). The untreated group had the largest RePu region. Compared with untreated group, the RAP (0) reduced the RePu area (p = 0.039). There was no significant difference between RAP (3), AVA (3) and AVA (6) groups and untreated groups. Although RAP (0) reduced the RePu area, the red RePu was still high and anemia still existed (Fig S1F). The results showed that monotherapy could not reduce the RePu area and did not improve anemia symptoms.

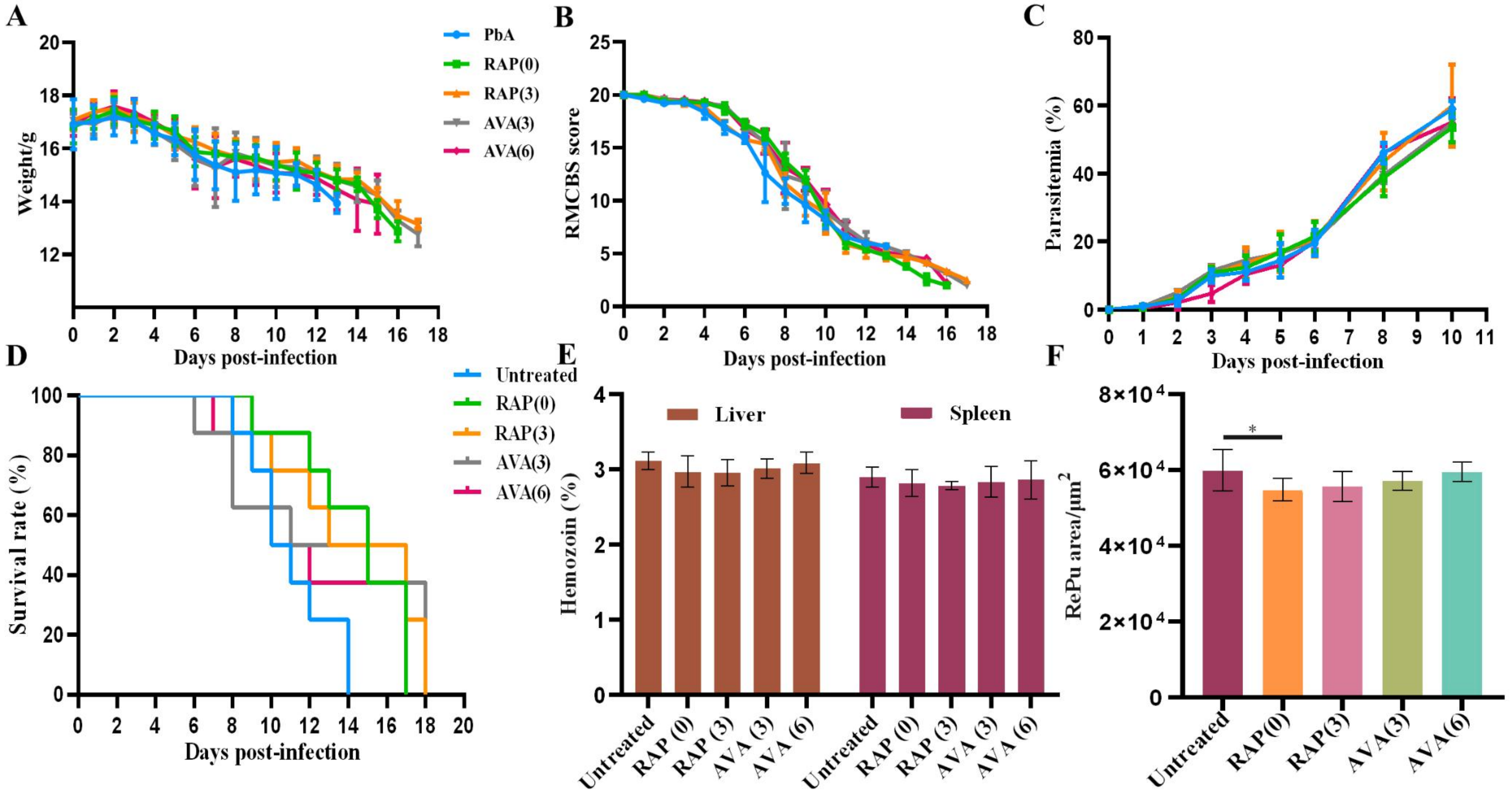

A
Weight/g
Days post-infection
PbA
RAP(0)
RAP(3)
AVA(3)
AVA(6)
B
RMCBS score
Days post-infection
C
Parasitemia (%)
Days post-infection
D
Survival rate (%)
Days post-infection
Untreated
RAP(0)
RAP(3)
AVA(3)
AVA(6)
E
Hemozoin (%)
Liver
Spleen
Untreated
RAP (0)
RAP (3)
AVA (3)
AVA (6)
F
RePu area/μm²
*
Untreated
RAP(0)
RAP(3)
AVA(3)
AVA(6)

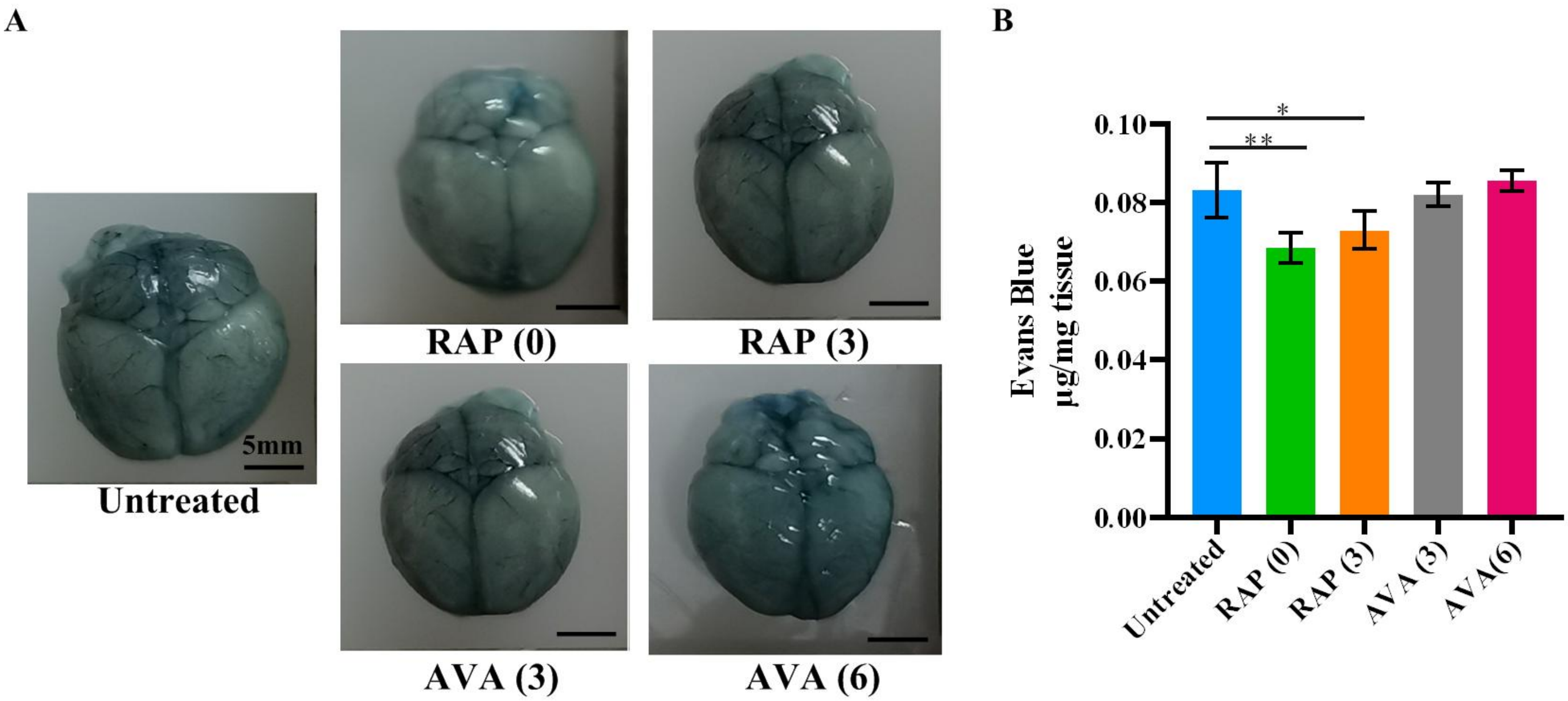

A
5mm
Untreated
RAP (0)
RAP (3)
AVA (3)
AVA (6)
B
Evans Blue
µg/mg tissue
0.10
0.08
0.06
0.04
0.02
0.00
*
**
Untreated
RAP (0)
RAP (3)
AVA (3)
AVA(6)

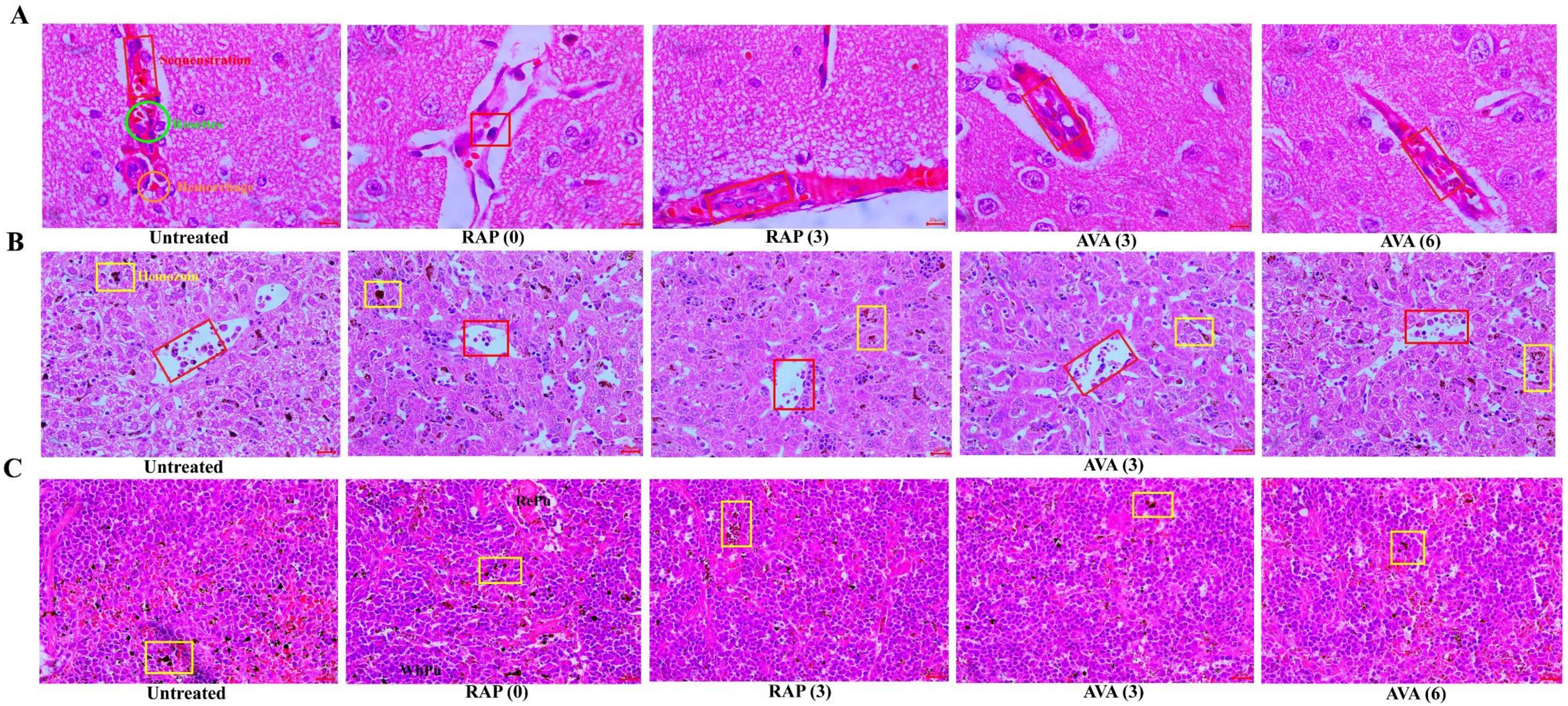
A
Sequenstration
Rosettes
Hemorrhage
Untreated
RAP (0)
RAP (3)
AVA (3)
AVA (6)
B
Hemozoin
Untreated
AVA (3)
C
RePu
WhPu
Untreated
RAP (0)
RAP (3)
AVA (3)
AVA (6)
